# Protective Effects of PACAP in Peripheral Organs

**DOI:** 10.3389/fendo.2020.00377

**Published:** 2020-07-14

**Authors:** Denes Toth, Edina Szabo, Andrea Tamas, Tamas Juhasz, Gabriella Horvath, Eszter Fabian, Balazs Opper, Dora Szabo, Grazia Maugeri, Agata G. D'Amico, Velia D'Agata, Viktoria Vicena, Dora Reglodi

**Affiliations:** ^1^Department of Forensic Medicine, MTA-PTE PACAP Research Team, University of Pécs Medical School, Pécs, Hungary; ^2^Department of Anatomy, MTA-PTE PACAP Research Team, University of Pécs Medical School, Pécs, Hungary; ^3^Department of Anatomy, Histology and Embryology, Faculty of Medicine, University of Debrecen, Debrecen, Hungary; ^4^Department of Biomedical and Biotechnological Sciences, Section of Human Anatomy and Histology, University of Catania, Catania, Italy; ^5^Heart Institute, Medical School, University of Pécs, Pécs, Hungary; ^6^Department of Drug Sciences, University of Catania, Catania, Italy

**Keywords:** PACAP, cytoprotection, periphery, apoptosis, ischemia

## Abstract

Pituitary adenylate cyclase activating polypeptide (PACAP) is a neuropeptide widely distributed in the nervous system, where it exerts strong neuroprotective effects. PACAP is also expressed in peripheral organs but its peripheral protective effects have not been summarized so far. Therefore, the aim of the present paper is to review the existing literature regarding the cytoprotective effects of PACAP in non-neuronal cell types, peripheral tissues, and organs. Among others, PACAP has widespread expression in the digestive system, where it shows protective effects in various intestinal pathologies, such as duodenal ulcer, small bowel ischemia, and intestinal inflammation. PACAP is present in both the exocrine and endocrine pancreas as well as liver where it reduces inflammation and steatosis by interfering with hepatic pathology related to obesity. It is found in several exocrine glands and also in urinary organs, where, with its protective effects being mainly published regarding renal pathologies, PACAP is protective in numerous conditions. PACAP displays anti-inflammatory effects in upper and lower airways of the respiratory system. In the skin, it is involved in the development of inflammatory pathology such as psoriasis and also has anti-allergic effects in a model of contact dermatitis. In the non-neuronal part of the visual system, PACAP showed protective effects in pathological conditions of the cornea and retinal pigment epithelial cells. The positive role of PACAP has been demonstrated on the formation and healing processes of cartilage and bone where it also prevents osteoarthritis and rheumatoid arthritis development. The protective role of PACAP was also demonstrated in the cardiovascular system in different pathological processes including hyperglycaemia-induced endothelial dysfunction and age-related vascular changes. In the heart, PACAP protects against ischemia, oxidative stress, and cardiomyopathies. PACAP is also involved in the protection against the development of pre-senile systemic amyloidosis, which is presented in various peripheral organs in PACAP-deficient mice. The studies summarized here provide strong evidence for the cytoprotective effects of the peptide. The survival-promoting effects of PACAP depend on a number of factors which are also shortly discussed in the present review.

## Introduction

**Figure 1:**
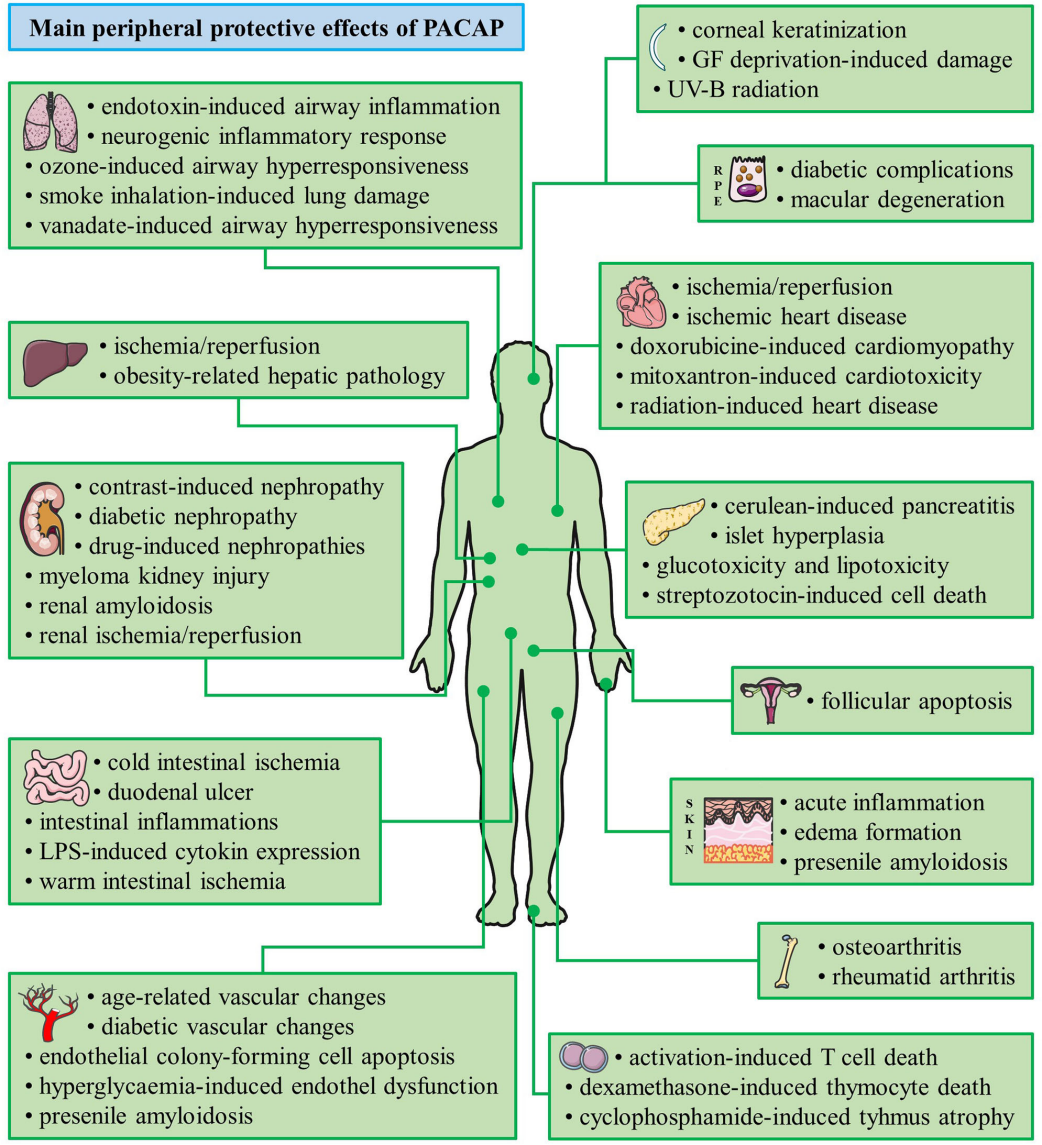
Summary of the main cytoprotective effects of PACAP. (Graphics are adapted from Servier Medical Art under a Creative Commons Attribution 3.0 Unported License.)

Pituitary adenylate cyclase activating polypeptide (PACAP) was discovered more than 30 years ago by Arimura et al. (1). The discovery was based on the ability of the hypothalamus-derived peptide to increase cAMP levels in cultured pituitary cells. Several studies following its isolation showed that PACAP exerts several distinct effects in the hypothalamo-hypophyseal system and other central regulatory pathways (2–7). PACAP belongs to the glucagon/secretin/vasoactive intestinal peptide family of peptides and it exists in two forms, with 38 and 27 amino acids. PACAP acts on G protein coupled receptors. The specific PAC1 receptor only binds PACAP, while the VPAC1 and VPAC2 receptors also bind vasoactive intestinal peptide with similar affinity (8–12). Early studies already pointed out the robust neuroprotective effects of PACAP *in vitro* and *in vivo* through a combination of antiapoptotic, antiinflammatory, and antioxidant effects (8–12). Neuroprotective actions have been shown, among others, in cerebellar granule cells, neuroblastoma cells, cortical neurons, and ganglionic cells against different toxic substances and harmful stimuli, mainly through the PAC1 receptors (9). *In vivo*, numerous animal models have been used to establish the potential neuroprotective effects of PACAP in pathological conditions (8–12). Although originally isolated in the central nervous system and early studies showed highest concentration in the brain, PACAP has a very widespread occurrence also in peripheral organs. Numerous studies have provided proof that PACAP exerts protective effects not only in the nervous system but also in many peripheral cell types and organs. The neuroprotective effects have been reviewed several times (8–16) but the peripheral protective effects of the neuropeptide have not been summarized in a review so far. Therefore, the aim of our present paper is to review the general cytoprotective effects of PACAP in non-neuronal cell types, peripheral tissues and organs (summarized in Figure 1, Tables 1, 2).

**Table 1:** *In vitro* studies showing protective effects of PACAP.

**Damaging insult/disease model**	**Cell line**	**References**
**INTESTINES**		
Oxidative stress	Human embryonic intestinal cells	(17)
**PANCREAS**		
Streptozotocin-induced cell death	Rat insulinoma	(18)
Cytokine-induced apoptosis	Mouse insulinoma	(19)
Gluco- and lipotoxicity	Mouse pancreatic beta cells	(20)
**LIVER**		
Oxidative stress	Mouse hepatocytes	(21)
Tumor necrosis factor-alpha/actinomycin D-induced apoptosis	Mouse hepatocytes	(21)
**KIDNEY**		
Oxidative stress	Rat kidney cells	(22)
Mineral oil evoked hypoxia	Mouse proximal tubular cells	(23)
Oxidative stress	Mouse kidney cells	(24)
CoCl2-induced hypoxia	Mouse kidney cells	(25)
Cisplatin toxicity	Mouse proximal tubular cells	(26)
Cisplatin toxicity	Human proximal tubular cells	(27)
Gentamicin toxicity	Human proximal tubular cells	(28)
Cyclosporine A toxicity	Human proximal tubular cells	(29)
Radiocontrast media toxicity	Human proximal tubular cells	(30)
Myeloma kappa-light chain toxicity	Human proximal tubular cells	(31)
Lipopolysaccharide-induced inflammation	Mouse podocytes	(32)
**RESPIRATORY TRACT**		
Cigarette smoke	Rat alveolar cells	(33)
**CORNEA AND PIGMENT EPITHELIAL CELLS**		
Hyperosmotic and oxidative stress (diabetic macular edema)	Human (adult) RPE cells	(34, 35)
UV-B exposure	Human corneal endothelial cells	(36)
Oxidative stress	Human (adult) RPE cells	(37, 38)
Hyperosmotic and oxidative stress-induced neovascularisation	Human (adult) RPE cells	(39)
Growth factor deprivation	Human corneal endothelial cells	(40)
Increased permeability (macular edema)	Human (adult) RPE cells	(41)
**IMMUNE CELLS AND THYMUS**		
UV irradiation	Peripheral T cells and T cell hybridomas	(42, 43)
Glucocorticoid-induced apoptosis	Rat thymocytes	(44)
**SKELETAL SYSTEM: CARTILAGE AND BONE**		
Oxidative stress	Chicken chondrogenic cells	(45)
Osteoarthritis	Rat chondrocytes	(46)
Oxidative and mechanical stress	Chicken chondrogenic cells	(47)
**CARDIOVASCULAR SYSTEM: VESSELS AND HEART**		
Oxidative stress6	Mouse hemangioendothelioma	(48)
TNF-α-induced apoptosis	Human endothelial cells	(49)
Ischemia/reperfusion	Rat cardiomyocytes	(50, 51)
Oxidative stress	Rat cardiomyocytes	(52, 53)

*RPE, retinal pigment epithelium; TNF, tumor necrosis factor*.

**Table 2:** *In vivo* studies showing protective effects of endogenous or exogenous PACAP.

**Damaging insult/disease model**	**Species**	**Exogenous or endogenous PACAP**	**References**
**INTESTINES**			
Mepirizole-induced duodenal ulcer	Rat	Exogenous	(54)
Small bowel cold ischemia	Rat	Exogenous	(55–57)
Small bowel cold ischemia	Mice	Endogenous	(58)
Small bowel warm ischemia	Rat	Exogenous	(56)
Small bowel warm ischemia	Mice	Endogenous	(59)
*T. gondii*-induced acute and subacute ileitis	Mice	Exogenous	(60, 61)
Dextran sulfate sodium-induced colitis	Mice	Endogenous	(62)
Inflammation-associated colorectal cancer	Mice	Endogenous	(63)
**PANCREAS**			
Cerulein induced-acute panreatitis	Mice	Endogenous	(64)
**LIVER**			
Warm liver ischemia	Mice	Both	(21, 65)
Obesity-induced liver steatosis	Mice	Exogenous	(66)
**SALIVARY AND OTHER EXOCRINE GLANDS**			
Salivary gland apoptosis	Snail	Exogenous	(67)
**URINARY SYSTEM**			
Warm renal ischemia	Mice	exogenous	(23, 68)
Warm renal ischemia	Rat	exogenous	(69–71)
Cisplatin-induced acute kidney injury	Mice	Exogenous	(26, 27)
Gentamicin-induced nephrotoxicity	Rat	Exogenous	(72)
Cyclosporine-A-induced nephrotoxicity	Mice	Exogenous	(29)
Contrast agent-induced nephropathy	Mice	Exogenous	(30)
Myeloma nephropathy	Rat	Exogenous	(31)
Streptozotocin-induced nephropathy	Rat	Exogenous	(73, 74)
Presenile kidney amyloidosis	Mice	Endogenous	(75)
Nephrotic syndrome	Zebrafish	Exogenous	(76)
**RESPIRATORY TRACT**			
Tracheal neurogenic inflammatory response	Rat	Exogenous	(77, 78)
LPS-induced subacute inflammation	Mice	Endogenous	(79)
Ozone-induced airway hyperresponsiveness	Rat	Exogenous	(80)
Ammonium vanadate-induced airway hyperresponsiveness	Guinea pig	Exogenous	(81)
Smoke inhalation-induced lung injury	Mice	Exogenous	(82)
**SKIN**			
Neurogenic skin edema	Mice	Endogenous	(83)
Oxazolone-hypersensitivity skin reaction	Mice	Endogenous	(84)
Presenile skin amyloidosis	Mice	Endogenous	(75)
Cornea and retinal pigment epithelial cells
Corneal keratinization	Mice	Both	(85, 86)
Physical corneal injury	Mice	Exogenous	(87, 88)
Physical corneal injury	Rabbit	Exogenous	(88)
RPE cells in diabetic retinopathy	Rat	Exogenous	(89)
**IMMUNE CELLS AND THYMUS**			
*S. aureus* enterotoxin B-induced T cell death	Mice	Exogenous	(42, 43)
Cyclophosphamide-induced thymus atrophy	Mice	Exogenous	(90)
**CARTILAGE AND BONE**			
Disturbed callus formation	Mice	Endogenous	(91)
Serum transfer-induced immune arthritis	Mice	Endogenous	(92)
**CARDIOVASCULAR SYSTEM**			
Ischemia/reperfusion	Pig	Exogenous	(93)
Diabetic vascular complications	Mice	Exogenous	(94)
Presenile vessel amyloidosis	Mice	Endogenous	(75)
Doxorubicin-induced cardiomyopathy	Mice	Endogenous	(95)
Mitoxantrone-induced cardiomyopathy	Mice	Exogenous	(96)
Irradiation-induced heart diseases	Mice	Exogenous	(97)

*RPE, retinal pigment epithelium; TNF, tumor necrosis factor*.

## Digestive System

### Intestines

PACAP has widespread expression in the gastrointestinal system (98–101). PACAP acts on different intestinal processes including motility (102), intestinal secretion of growth factors (103), and activity of interstitial Cajal cells (104). *In vitro* investigations of PACAP in small intestine were carried out using INT407 cells originally obtained from human embryonal jejunum and ileum (17). PACAP showed protective effects against oxidative stress, but it was not effective in CoCl_2_-induced *in vitro* hypoxia. Surprisingly, if cells were exposed to gamma irradiation, PACAP acted negatively on clone-forming ability, but this might be due to a function in reducing the number of damaged cells (17). Furthermore, *Adcyap1* small interfering RNA transfection led to higher vulnerability in INT407 cells suggesting a protective role of endogenously present PACAP (17).

*In vitro* experiments using HCT-8 human colonic tumor cells revealed proliferation-enhancing effect of PACAP (105). The authors detected PACAP and specific PAC1 receptor in the HCT-8 cell line. In addition, PACAP-38 was shown to suppress Fas receptor, suggesting a possible role of PACAP in cell survival (105). Lelievre et al. tested the effect of PACAP-27 in four human colonic adenocarcinoma cell lines (HT29, SW403, DLD-1, Caco-2). They found that long-term treatment with PACAP or VIP reduced cell proliferation (106). Bacterial adhesion plays a crucial role in gastrointestinal infections. Illes et al. (107) examined the effect of PACAP on bacterial adhesion in small and large intestinal cell lines. PACAP influenced colony numbers of investigated bacteria neither in small intestinal INT407 nor in large intestinal Caco-2 cells. On the other hand, PACAP was able to act on expression of certain cytokines: it induced IL-8 and CXCL-1 activation (107).

Previous studies aimed to investigate the possible effect of PACAP in different models of intestinal pathologies. Protective effect of PACAP in a rat model of duodenal ulcer was investigated by Yagi et al. (54). Rats treated with mepirizole showed increased gastric acid secretion and hemorrhagic lesions in proximal duodenum. The applied intravenous PACAP-27 treatment led to increased HCO_3_- secretion thus it could significantly reduce the severity of duodenal lesions with no effect on gastric acid secretion (54). Other studies have targeted to explore the effect of PACAP in ischemia/reperfusion. Ferencz et al. (55–57) described protective effect of PACAP in a rat model of small bowel autotransplantation, modeling cold ischemia injury. Small bowel was removed and stored in standard preservation solution with or without additional PACAP. The histological damage caused by cold ischemia was ameliorated by PACAP: changes in mucous layer were reduced and crypt morphology was better preserved (55). Besides preserving the morphology, authors found that PACAP did not change lipid peroxidation but kept the endogenous scavanger capacity. Effects of endogenously present PACAP against cold ischemic injury were investigated using PACAP deficient mice (58). Cold preservation injury was established with removing small bowel from PACAP deficient and wild type animals. Histological analysis showed more severe destruction of mucous, submucous layers, and crypts in PACAP deficient mice compared to wild type animals (58). Furthermore, the effect of endogenous PACAP was also tested in warm intestinal ischemia (59). Warm intestinal ischemia was evoked by occlusion of the superior mesenteric artery. The intestinal injury indicated by tissue damage was more severe in case of mice lacking PACAP. Oxidative stress markers, like malondialdehyde have also shown significant differences between PACAP deficient and wild type animals (59).

Protective actions of PACAP were also studied in small and large intestinal inflammations (98). Heimesaat et al. (60) described protective effect of PACAP against Toxoplasma gondii-induced acute ileitis. Both PACAP prophylaxis and treatment were effective, mice obtaining PACAP prophylaxis or treatment showed a higher survival rate (60). Authors have extended their experiments in order to investigate whether PACAP could alleviate subacute ileitis induced by low-dose Toxoplasma gondii in mice having human gut microbiota (61). PACAP treatment led to less distinct apoptotic responses in ileal and colonic epithelia. Furthermore, not only intestinal but extraintestinal sequelae of low-dose Toxoplasma gondii infection were suppressed (61).

Effect of PACAP in large intestinal inflammation can be presumed from changes of PACAP immunoreactivity in different large intestinal pathologies in the pig (108). A considerable upregulation of PACAP mRNA level and downregulation of VPAC1 receptor were detected in transient receptor potential Ankyrin type 1 (TRPA1) knockout mice in DSS-induced colitis (109). In addition, PACAP expression was significantly reduced in transient receptor potential cation channel subfamily V member 1 (TRPV-1) knockout mice, which together with reduced expression of VIP can contribute to local pro-inflammatory environment in these animals (110). Horvath et al. (98) investigated the possible role of PACAP in human inflammatory bowel diseases. PACAP expression was significantly higher in samples obtained from patients suffering from ulcerative colitis, while this increase could be suppressed by antibiotic therapy. Role of endogenously present PACAP was tested in dextran sulfate sodium (DSS)-induced colitis by two research groups (62, 63). Azuma and colleagues found, based on the histological analysis and the determination of disease activity index of PACAP knockout mice, that mice lacking PACAP had a significantly higher vulnerability than wild type controls (62). Investigations of Nemetz and coworkers supported these findings. Mice lacking PACAP displayed more severe symptoms of colitis and significantly stronger colonic inflammation. Moreover, 60% of DSS-treated PACAP deficient mice developed aggressive-appearing colorectal cancer (63). An altered microbiota composition can also be in the background of the increased vulnerability of PACAP knockout mice, as investigations of intestinal microbiota composition in wild type and PACAP deficient mice showed that Bifidobacteria were virtually absent in PACAP deficient mice, even when they were still breastfed (111).

### Pancreas

PACAP is present in both the exocrine pancreas and in the endocrine islets of Langerhans and it is thought to be a potent intra-pancreatic regulator of beta cells under physiological and pathological conditions (112–114). Interestingly, cerulein-induced pancreatitits was aggravated in PACAP deficient mice (64), but pancreatic beta cells derived from rat insulinoma, key elements in pathogenesis in diabetes mellitus, were prevented from streptozotocin-induced cell death (18). Han and Wu (19) found that Adcyap1 overexpression reduced cytokine-induced apoptosis in a mouse insulinoma cell line. Moreover, pancreatic islets prepared from PACAP knockout and wild type mice showed significant differences in defense against glucotoxicity and lipotoxicity (20). Pancreatic islets cultured with high glucose or palmitate displayed severely impaired glucose-induced first phase Ca^2+^ increase and insulin secretion in PACAP deficient mice, but not in wild type animals (20). PACAP overexpression in KKAγ mice suffering from diabetes type II attenuated hyperinsulinaemia and islet hyperplasia without alteration of plasma glucose, glucose tolerance and insulin tolerance (115). This was observed both in animals on normal diet and in mice kept on high-fat diet suggesting that PACAP regulates abnormal increase in islet mass and hyperinsulinaemia in type II diabetes (115, 116).

### Liver

Few data indicate that PACAP is also protective in some pathological liver conditions. Although no effects on survival were reported in normal or tumorous human hepatocyte cells *in vitro* exposed to oxidative stress (22), PACAP showed protection in mouse hepatocytes *in vitro* exposed to oxidative stress by H_2_O_2_ or subjected to apoptosis-inducing TNF-α/actinomycin D treatment (21). *In vivo* protection was also described in ischemia/reperfusion liver injury (21). Pretreatment with PACAP27 or 38 1 h before the onset of ischemia diminished serum alanine aminotransferase levels, reduced the accumulation of neutrophils and macrophages, suppressed inflammatory chemokines (CXCL-1, CCL-2, CXCL-10) and cytokines (TNF-α, IL-1β, IL-6, and IFN-β). The histological structure of the liver was better preserved after PACAP treatment: necrosis and apoptosis was reduced, caspase-3 activity was decreased along with increased antiapoptotic molecule expression of bcl-2 and bcl-xL via cAMP/PKA activation. The phosphorylation, thus activation, of IκBα/NF-κB p-65 proteins was reduced and toll-like receptor four immune response was inhibited (21).

The role of endogenous PACAP in liver protection was examined in PACAP deficient mice. Ischemia/reperfusion injury was augmented in animals lacking endogenous PACAP: serum alanin aminotransferase levels were increased and more severe tissue damage, indicated by edema, hemorrhage, congestion, and hepatocellular necrosis, was observed compared to wild type mice (21). In wild type mice, ischemic injury followed by reperfusion led to a transient drop in endogenous PACAP mRNA expression followed by a progressive increase. Similarly, ischemic reperfusion injury triggered changes in the receptor expression: VPAC receptor expression was also increased after an initial drop, while PAC1 receptor expression was increased from the onset of the ischemic period (21). A follow-up study demonstrated that the protective effects of PACAP in hepatic ischemia/reperfusion are partially mediated by induction of Yes-associated protein, a cellular modulator of tissue regeneration (65). The ischemia-induced induction of this protein was absent in mice lacking endogenous PACAP, while PACAP substitution enhanced its expression.

A recent paper showed that PACAP can alleviate inflammation and steatosis, thus it can be protective in obesity-related hepatic pathology and can ameliorate glucose and lipid metabolism (66). These effects were mainly mediated by the specific PAC1 receptor, involving Fas apoptosis inhibitory molecule (FAIM), proven both *in vitro* and *in vivo*. Lower PACAP expression was found in leukocytes isolated from obese human patients, and levels were lower in livers of obese mice. PACAP treatment of obese mice not only increased FAIM levels, but also decreased serum triglycerides and total cholesterol and reduced body weight (66). Liver triglycerides were also reduced after PACAP treatment. Systemic inflammatory markers were decreased, including MCP-1, IL-6, and TNF-α. Examining fat tissue revealed that PACAP treatment reduced the size of adipocytes and attenuated liver steatosis. Altogether, these data indicate that PACAP ameliorates hepatic metabolism and inflammation in obesity (66). An indirect mechanism has also been suggested to play a role in the regeneration-stimulating effect of PACAP: the PACAP-regulated selenoproteine expression is strongly stimulated in liver cells during the regenerative process that occurs after partial hepatectomy (117).

### Salivary and Other Exocrine Glands

Occurrence of PACAP and receptors has been shown in several exocrine glands and their secretions, including salivary, mammary and lacrimal gland (85, 114, 118–121). Among others, PACAP enhances salivary and lacrimal gland secretion, increases salivary gland blood flow, and is implicated in breast cancer growth (85, 118, 122–125). In contrast to the general cytoprotective effects of PACAP, in MCF-7 breast cancer cell line PACAP induced reactive oxygen species through H_2_O_2_ production, induced calcium release, and promoted apoptosis by increasing Bax and decreasing bcl-2 expression (126). The evolutionarily conserved nature of the antiapoptotic effect has been proven in molluscan salivary gland (67). PACAP induced a significant elevation of cAMP level in salivary gland extracts and attenuated the apoptosis-inducing effect of dopamine and colchicine, shown by the reduced caspase-positive cells (67).

## Urinary System

Widespread distribution of PACAP and its receptors has been described in the kidney and lower urinary tract (127, 128), where PACAP plays distinct roles in the micturition pathways, blood supply, hormone production and inflammation (128–130). Direct protective effects of PACAP have mainly been described in the kidney, its nephroprotective actions have been widely studied. Its protective effects could be observed in different models of renal pathological conditions (131). It shows protective effects both *in vitro* and *in vivo*. *In vitro* data are available proving the renoprotective effect of PACAP in different models of cellular damage. It was shown to decrease the cell survival worsening effect of oxidative stress in primary renal cell cultures (22). In addition, Li et al. described its protection against mineral oil evoked *in vitro* hypoxia in proximal tubule epithelial cells obtained from wild type and MyD88 deficient mice (23). Furthermore, its endogenous action against oxidative stress and hypoxia can also be observed using PACAP deficient mice, responding with higher vulnerability to oxidative stress leading to decreased survival rate (24). Similarly, this susceptibility could also be detected when renal cells were exposed to CoCl_2_-evoking *in vitro* hypoxia (25). Experiments investigating the effect of PACAP against proteinuria mimicking albumin treatment in human proximal tubule cells showed that PACAP could not influence cell viability either positively or negatively (132). It was not able to change the increased TGF-β1 expression either.

*In vivo* observations were obtained from a series of experiments modeling renal ischemia/reperfusion injury. The first experiments proving PACAP's renoprotective effect in ischemia/reperfusion were performed by Riera et al. (69). They found that continuous PACAP infusion improved renal function, attenuated morphological damage, and influenced inflammatory cellular infiltration. In addition, Szakaly et al. performed experiments, in which PACAP was able to ameliorate tubular damage and the level of oxidative stress, thus to decrease mortality of rats that underwent ischemia/reperfusion (70). PACAP was shown to reverse the cytokine expression profile after ischemic injury (133). Such renoprotective effect was also detected in mice (23, 26, 27). Khan et al. (68) performed experiments to investigate the involvement of toll-like receptors. Dozens of toll-like receptor genes changed after ischemia, while PACAP was able to reverse these changes. Gender-dependence was investigated in a recent study comparing renoprotective effect of PACAP in male and female rats (71). Tubular alteration was markedly less severe in female rats. Female animals showed better results in both PACAP-treated and vehicle-treated experimental groups indicating the presence of several additional protective factors in females.

Actions of PACAP have also been studied in different models of drug-induced nephropathies. PACAP was able to diminish the nephrotoxic effect of gentamicin both *in vitro* and *in vivo*. *In vitro* experiments showed its cell survival enhancing effect in human proximal tubule cell line (HK-2) assessed by cytotoxicity assay (28). In the same experimental model, it could counteract the downregulating effect of gentamicin on dipeptidyl peptidase IV and vascular endothelial growth factor (28). In accordance with *in vitro* data, *in vivo* investigations have also explored its protective effect in gentamicin-induced nephropathy. Tubular damage caused by the accumulation of gentamicin could be attenuated by repeated intravenous administration of PACAP in rats, as indicated by the decreased TNF-α production (72). Chemotherapeutic agents, like cisplatin, can also lead to renal injury (134). *In vitro* studies using human HK-2 cells proved that PACAP protected against cisplatin-induced injury, decreased the cisplatin-induced TNF-α activation, influenced signaling pathways activated by cisplatin (27). Li et al. examined primary mouse renal proximal tubular epithelial cell culture (26). In accordance with results in human cells, PACAP's protective effect was detectable. *In vitro* results were supported by *in vivo* renoprotection in a mouse model of cisplatin-induced nephrotoxicity (26). Mice treated with PACAP showed less severe decrease of renal function. Furthermore, PACAP treatment was able to alleviate morphological damage and reverse the cisplatin-induced p53 activation. A further drug, cyclosporine A has also been widely studied. Cylosporine A, a potent immunosuppressant used for preventing allograft rejection and in treatment of autoimmune diseases, can also lead to impaired renal function (135). Khan et al. (29) investigated the effect of PACAP against cyclosporine A both *in vitro* and *in vivo*. PACAP was able to improve morphological changes and attenuate TGF-β activation caused by cyclosporine A treatment in HK-2 cells. *In vitro* data were further supported by their *in vivo* findings. PACAP could improve the impaired renal function with normalizing serum creatinine level. In addition, tubulointerstitial damage was diminished and changes in cell junctional markers were restored by PACAP treatment (29). Protective effect of PACAP against contrast-induced nephropathy was also tested by Khan et al. (30). PACAP could decrease the proliferation-inhibiting effect of both ionic and non-ionic contrast media in HK-2 cells. If added prior to urografin, it was able to enhance cell survival of HK-2 cells. Furthermore, PACAP decreased the elevated kidney injury molecule-1 (KIM-1) expression evoked by contrast medium (30). *In vivo* findings complete the *in vitro* data. Mice pretreated with PACAP before contrast agent did not show severe tubular damage, apoptosis, increased oxidative stress or inflammatory reactions unlike animals receiving only contrast agent. A kidney biomarker assay further supported these data, as numerous markers associated with kidney injury were decreased in PACAP-treated mice (30).

Arimura et al. studied the actions of PACAP in myeloma kidney injury both *in vitro* and *in vivo* (31). *In vitro* myeloma kidney injury was generated with kappa light chains isolated from the urine of a patient suffering multiple myeloma. Human renal proximal tubule cells exposed to kappa light chains were protected by PACAP: it could mitigate the cellular injury and elevated expression of IL-6 and TNF-α. *In vivo* investigations in rats were in accordance with *in vitro* results. Rats receiving both PACAP and myeloma light chain showed reduced cytokine activation compared to animals treated only with light chain (31). In addition, effectiveness of PACAP was also examined in a single patient case study, in which it was shown to reduce free lambda light chains in urine indicating its possible therapeutic use in the future (136). Another target of investigations exploring renoprotective effect of PACAP is diabetic nephropathy, the leading cause of renal insufficiency (137). Sakamoto et al. used podocytes to model inflammation in diabetic nephropathy (32). PACAP was able to reduce the lipopolysaccharide-induced proinflammatory cytokine activation, ERK phosphorylation and NF-κB transnuclear localization. *In vivo* studies revealed protection in streptozotocin-induced diabetes in rats (73). Intraperitoneal PACAP treatment led to less severe morphological changes and reduced proinflammatory cytokine activation (73). Li et al. (72) applied continuous PACAP infusion for 2 weeks and they found that PACAP reduced the diabetic changes like proteinuria and glomerular enlargement. Molecular mechanism of PACAP-exerted protective effects was also examined in streptozotocin-induced diabetes (74). Results showed that PACAP could decrease the activation of apoptotic signaling pathways.

Investigating PACAP deficient mice, it was revealed that animals lacking endogenously present PACAP suffer from presenile systemic amyloidosis (75). Severe amyloidosis can be observed in several organs including the kidney. Kidney was one of the most affected organs, amyloid deposits were found in renal corpuscles. Level of renal function was also in accordance with amyloid deposits: serum creatinine level was increased in aging PACAP-deficient mice (75, 138).

PACAP suppression experiments in zebrafish of Eneman et al. revealed that nephrin depletion, a model of nephrotic syndrome is associated with adcyap1a and vip downregulation (76). Using adcyap1a and adcyap1b morpholinos the authors decribed more severe sequelae of nephrin depletion. In addition, administration of human PACAP38 could rescue the phenotype of zebrafish embryos injected with PACAP morpholino, but it was not able to save them in case of nephrin depletion. Nephrotic syndrome was also modeled by adriamycin exposure, when only adcyap1a gene was downregulated. Furthermore, nephrotic fishes showed reduced protein expression of PACAP. PACAP morpholinos worsened the change in phenotype induced by adriamycin exposure, which could be attenuated by addition of human PACAP38 (76).

## Respiratory Tract

Although the role of PACAP in the respiratory tract is not as widely studied as that of VIP, PACAP and is receptors occur in the entire length of the respiratory tract (139–141) and several effects have been described in airway smooth muscle contraction and mucous secretion (142–144). It has been reported that PACAP increases allergic reactions in the human nasal mucosa by increasing resistance and plasma leakage (145), but PAC1 receptor is implicated in anti-inflammatory reactions and mediates alleviation of bronchial hyperreactivity (146). Exogenous PACAP diminished both capsaicin- and electric field stimulation-evoked sensory neuropeptide release in a concentration-dependent manner in trachea preparations (77, 78), showing that PACAP is able to diminish neurogenic inflammatory response *in vivo*. The protective role of PACAP has also been demonstrated in a lung inflammation model of mice (79). In endotoxin-induced subacute airway inflammation, airway hyperreactivity, histopathological changes, and myeloperoxidase activity were markedly higher in mice lacking endogenous PACAP, pointing to the anti-inflammatory role of endogenous PACAP in the lungs (79). In another rat model, in vanadate-induced airway hyperresponsiveness, PACAP inhalation alleviated the increase in bronchial resistance, reduced the increased inflammatory chemokine, and cytokine release and improved the antioxidant status, also pointing to the potential of PACAP treatment in inflammatory and allergic respiratory conditions (80). Similar results were found in ozone-induced airway hyperresponsiveness, which was suppressed by PACAP without affecting plasma extravasation (81). Furthermore, Yu et al. (82) have described that PACAP, bound to a traversing-enhancing TAT peptide, can alleviate smoke inhalation-induced condition. They found that both PACAP and PACAP-TAT decreased mortality, led to a body-weight increase, alleviated edema and vascular permeability increase, and decreased oxidative stress as indicated by reduced myeloperoxidase activity, interleukin-6, and malondyaldehyde levels while increased catalase levels in the lungs of mice that were exposed to repeated smoke inhalation (82). PACAP and PACAP-TAT treatments also resulted in decreased cell infiltration and bronchial epithelial hyperplasia. These data indicate that PACAP can alleviate smoke inhalation-induced damage of the lungs. These data confirmed earlier results showing that PACAP protected rat alveolar L2 cells from cytotoxicity of cigarette smoke by reducing caspase activity resulting in reduced apoptotic cell death (33). PACAP has also been implicated in lung cancer cell growth (147). PACAP stimulates colony formation and nuclear oncogene expression in NCI-N417 lung cancer cells, while PACAP antagonist treatment slows down small cell lung cancer growth (148, 149) and lower levels of PACAP were described in human lung cancer biopsies in comparison with neighboring healthy tissue (150). PACAP induces vasodilation, including pulmonary vessels. The absence of its specific receptor PAC1 causes pulmonary hypertension and right heart failure after birth (151). These findings demonstrate the crucial importance of PAC1-mediated signaling for the maintenance of normal pulmonary vascular tone during early post-natal life (151).

## Skin

The presence of PACAP and receptors has been shown in the skin (83, 152, 153). Potent vasodilatory and edema-building effects have been attributed to cutaneous PACAP treatment soon after its discovery (154, 155). Recent studies have shown that PACAP stimulates sweat gland activity (156). PACAP in the skin is implicated as a protective factor in the development of inflammatory dermatological conditions, such as psoriasis (153, 157) and neurogenic skin inflammation (83). Although both stimulatory and inhibitory actions on skin edema formation have been described, PACAP deficient mice show increased delayed-type of hypersensitivity reaction induced by oxazolone (84). Mice lacking endogenous PACAP had increased edema formation, moderately enhanced cellular inflammatory reactions, and increased levels of the inflammatory cytokine monocyte chemoattractant protein-1 levels (84). These results point to the anti-allergic effects of PACAP in a model of contact dermatitis. We have already mentioned above the presenile appearance of a systemic type of amyloidosis in PACAP deficient mice (75, 138). The amyloid deposition was markedly present in the skin, under the epidermis. In the skin, the main location of the deposits was the dermal papillary layer, continuous with the homogeneous mass in the connective tissue surrounding appendages (hair follicles and sebaceous and sweat glands) and vessel walls. While in wild type mice occurrence of amyloidosis was observed in 57% of the animals at old age, and only 14% young mice, already in 67% of young PACAP KO mice and nearly 90% of old PACAP KO mice exhibited amyloid deposits (75) in the skin. This shows that PACAP deficiency can be an attributing factor in skin aging.

## Non-neuronal Parts of the Visual System: Cornea and Retinal Pigment Epithelial Cells

The effects of PACAP in the visual system have been widely investigated and numerous reports have shown the effects of PACAP in the neural parts, especially in the retina. Both *in vitro* and *in vivo* studies have demonstrated that PACAP is a potent neuroprotective agent in different retinal pathologies. These effects have been reviewed in several papers (86, 158, 159). Other, non-neuronal parts of the eye have also been investigated for the actions of PACAP. Among others, PACAP influences sphincter and dilator pupillary muscle contractility (160), increases cAMP in the ciliary epithelium (161) and is involved in ocular inflammatory reactions (162). Regarding cytoprotective effects, several studies have described that PACAP protects the cornea. The cornea consists of 5 layers, the outer epithelium building the barrier toward the outside and the inner endothelium serves as a barrier toward the aqueous humor. Both inner and outer epithelial layers play an important role in maintaining the hydration and transparency of the main stromal layer, which is separated from the outer and inner epithelial layers by outer and inner limiting membranes, respectively. PACAP receptors have been described on the surface of the cornea (85). It is thought that the peptide produced by the lacrimal gland and present in the lacrimal fluid can act on the surface of the cornea and can play a role in the regeneration of the corneal surface epithelial cells. Indeed, PACAP eye drops prevent corneal keratinization in PACAP deficient mice (85). A PACAP-derived peptide has been shown to promote corneal wound healing (87, 88). PACAP given in form of eye drops not only acts directly on the surface but also passes ocular barriers and is able to induce protective effects in the retina (163, 164).

Recent studies have reported that PACAP is protective in corneal endothelial cells, where PACAP and all three receptors are expressed (34, 35). In isolated human corneal endothelial cells PACAP protected growth factor deprivation-induced decrease in cellular viability and restored transepithelial electrical resistance (34). Furthermore, PACAP increased the expression of tight junction proteins and stimulated corneal endothelial repair demonstrated in a wound healing assay (34). Noteworthy, PACAP exerted a protective effect on corneal endothelium against ultraviolet B (UV-B) radiation, by reducing the activation of apoptotic pathway through a down-regulation of bax and cleaved caspase-3 and up-regulation of bcl-2 protein. Moreover, PACAP preserved corneal endothelium integrity following UV-B exposure by increasing transepithelial electrical resistance and the expression of ZO-1 and claudin-1 proteins (36). The same authors investigated the involvement of epidermal growth factor receptor involvement in PACAP-induced protection of corneal endothelial cells (35). They found that PACAP, through PAC1 receptor, induced epidermal growth factor receptor phosphorylation and MAP kinase/ERK1/2 activation. These results are also in accordance with data obtained in retinal pigment epithelial cells where PACAP restored cell viability in pigment epithelial cells exposed to different stressors, induced ERK and epidermal growth factor receptor phosphorylation and ameliorated junctional protein damage (37–40).

The retinal pigment epithelium is the outermost, non-neural layer of the retina. The apical surface of the cells is in contact with the outer segments of the photoreceptor cells, while the basolateral surface attaches to the Bruch's membrane, which separates the pigment cells from the choriocapillary layer. Tight junctions interconnecting retinal pigment cells are the main components of the outer blood-retina barrier, which has essential roles in the maintenance of the retinal homeostasis. Dysfunction of the tight junctions has been observed in diabetes and ischemia, leading to leakage of macromolecules into the retina, contributing to the development of retinopathies (165). Retinal pigment epithelial cells are likely to undergo hyperosmotic stress during the development of macular edema, resulting in reduction in aquaporin-4 expression (166). Moreover, hyperosmolarity was observed to induce transcription of bFGF and HB-EGF genes and secretion of bFGF from the pigment cells (167). One of the most important factors secreted by the pigment epithelial cells is vascular endothelial growth factor, VEGF (168–172). Overexpression of VEGF is one of the major inducers of age related macular degeneration and proliferative diabetic retinopathy, which are among the leading causes of blindness worldwide (173).

All three PACAP receptors (PAC1, VPAC1, and VPAC2) mRNAs were detectable in the pigment epithelium (174). According to Mester et al. (37) adult retinal pigment epithelial cells (ARPE-19) exposed to H_2_O_2_ could be rescued with PACAP in a dose-dependent manner. In a subsequent study it was also proved that PACAP could inhibit the expression of pro-apoptotic factors (Bad, Bax, and Hif1α) and elevate the levels of anti-apoptotic factors such as ERK1/2 and CREB (38). Besides its general cell protective, pro-survival, and anti-inflammatory effects, PACAP possesses protective effects on tight junctions of endothelial and epithelial cells (175). PACAP was shown to have a protective effect on the barrier properties of the cells of the outer-blood-retinal barrier in the presence of factors accounting for diabetic macular edema (34, 35, 41, 89).

PACAP could also reduce the concentration of VEGF in ARPE-19 cells. Moreover, PACAP was able to attenuate the levels of some other pro-angiogenic proteins (uPA, angiogenin, and endothelin-1). As a conclusion, PACAP is among the emerging molecules to fight diabetic complications and macular degeneration, similarly to VEGF antagonists, antioxidants, anti-inflammatory agents, and other neuropeptides (39).

## Immune Cells and Thymus

PACAP is a well-established modulator of innate and acquired immunity and exerts protective functions in immunological diseases, although the presence of both anti- and pro-inflammatory roles of PACAP depending on the immune status, disease, age, and pathological conditions complicate the immunological role of PACAP (176, 177). As there are several reviews on the immunomodulatory roles of PACAP (177–179), the present review summarizes only data regarding direct cellular protection in lymphatic organs and cells. Activation induced cell death in T lymphocytes is an important mechanism in peripheral tolerance, initiated by antigen reengagement, and mediated through Fas/Fas ligand (FasL) interactions. PACAP was found to inhibit this induced cell death *in vivo* and *in vitro* in peripheral T cells and T cell hybridomas (42, 43). Both forms of PACAP, PACAP27 and PACAP38, can protect CD4+CD8+ thymocytes from glucocorticoid-induced apoptosis, suggesting an involvement of PACAP in thymic T-cell maturation (44). The expression of PAC1 receptor and PACAP increased in the degenerative thymus induced by cyclophosphamide (90). The authors have also described that while high dose PACAP had no protective effects against cyclophosphamide-induced thymus atrophy, low dose PACAP promoted the thymus index, inhibited apoptosis, enhanced oxidative status, and decreased caspase activity (90).

## Skeletal System: Cartilage and Bone

Neuropeptides have important functions in the development of skeletal elements and also in regeneration processes (180, 181). Detailed analysis of PACAP receptors has been performed in chondrogenic cell cultures (45) and osteogenic cell lines (182, 183). The specific PAC1 receptor has been identified in bone and cartilage (45, 183) and the activation of PAC1 by PACAP has positive effect on cartilage and bone formation (45, 184) via the activation of protein kinase A (PKA)-regulated pathways. PACAP also positively regulates matrix expresssion of both musculoskeletal elements. Addition of PACAP to chondrogenic cell cultures increases the activation of Sox9 (SRY-related HMG-BOX gene) and regulates the expression of HAS2 and HAS3 (hyaluronan synthase) expression (45). In bone PACAP increased the activation of alkaline phosphatase (ALP) and elevated the expression of collagen type I (184).

In degenerative cartilage diseases increasing number of experiments have been performed in the last decade to prove that PACAP plays a protective role. First, it was shown that the expression of PAC1 receptor was altered in specific layers of cartilage in osteoarthritis (OA) (46) and reduced during oxidative stress (45). On the other hand, the neuropeptide has a preventive function in pathological processes. In cartilage, the antagonist PACAP6-38 behaves as an agonist and administration of both PACAP1-38 and 6-38 protected against the harmful effect of oxidative stress in high density chondrogenic cell cultures via the elevation of collagen type II, aggrecan and hyaluronic acid expression (45). PACAP elevated the activation of PP2B (protein phosphatase 2B) and subsequently triggered the activation of NFAT (Nuclear factor of activated T-cells). In oxidative stress PACAP administration prevented the phosphorylation processes of chondrogenic signaling pathways in chicken high density cultures. The administration of the neuropeptide was able to defend cartilage formation during mechanical overload via decreasing the expression of collagen type X, a characteristic sign of OA cartilage. On the other hand, it increased collagen type II expression inducing a chondrogenic phenotype formation (185). PACAP signaling also communicates with the hedgehog pathways leading to decreased expression of SHH (sonic hedgehog) and IHH (Indian hedgehog) (185). The reduced activation of hedgehog pathways kept cartilage in a prehypertrophic condition (185) suggesting an important role of PACAP in cartilage degradation processes. Furthermore, mechanical overload and presence of reactive oxygen species trigger the activation of several matrix degrading enzymes such as matrix metalloproteinases, hyaluronidases, and aggrecanases in OA. PACAP was able to reduce the activation of matrix metalloproteinases and aggrecanases in chondrogenic cell cultures exposed to forced physical stress and oxidative stress, further strengthening the preventive function of the neuropeptide in cartilage diseases (47). In OA patients concentration of PACAP is decreased (186) and the administration of PACAP with hyaluronic acid injection increased the synovial fluid PACAP concentration and prevented the cartilage degradation processes (186). Moreover, PACAP addition during anterior cruciate ligament related OA formation can be an adjuvant therapy to prevent the physiological structure of articular cartilage (187). Taken together application of PACAP as a potential therapeutic target of OA formation is likely as it was discussed by Grassel et al. (188).

Some data point to the possible importance of PACAP in bone regeneration processes. First of all, the presence of PACAP is needed for proper bone architecture formation (184). In the lack of PACAP the long bones are more fragile and their organic and inorganic extracellular matrix balance and distribution are disturbed (184). In callus formation PACAP is proven to have important function via the activation of BMP (bone morphogenetic protein) signaling pathway and balance the ALP expression (91). In mice lacking endogenous PACAP, callus formation after tibia fracture was disturbed with several alterations in compensatory pathways (91). Furthermore, PACAP has an important function in bone turnover and inflammation (92). On the other hand, the presence of PACAP inhibited osteophyte formation and had a preventive role in rheumatoid arthritis development (92).

## Cardiovascular System: Vessels and Heart

PACAP is a well-known vasodilator peptide (189–191). Receptors are found in vessel walls and PACAP-induced vasodilatory effects have been demonstrated in various vessels *in vitro* and *in vivo*. This perfusion-increasing potency has beneficial effects in several organs, but can also trigger migraines through this activity in meningeal arteries (192, 193). PACAP has also been described to preserve post-ischemic cerebrovascular reactivity in pigs, independent of its vasodilatory effect (93). As there are several original and review papers published in the recent years regarding this vasodilatory effects of PACAP (191–196), now we focus more on the protective effects of PACAP exerted directly on the vascular wall, especially on endothelial cells. First results showed protective effects against oxidative stress: exposure of mouse hemangioendothelioma cells to hydrogen peroxide resulted in a robust reduction of viability and an increase of apoptotic cells, while co-incubation with PACAP increased cell viability and reduced the number of apoptotic cells (48). PACAP treatment also ameliorated the reduced level of ERK phosphorylation and counteracted the increased phosphorylation of the pro-apoptotic JNK and p38 MAP kinases. PACAP also exerts a cytoprotective effect on endothelial colony forming cells exposed to TNF-α and partially rescues their proliferation potential inhibited by prolonged TNF-α exposure (49). In a more recent study, ameliorating effect of PACAP has been described in hyperglycaemia-induced endothelial dysfunction, an important factor contributing to diabetes-related vascular pathology (94). PACAP reduced the hyperglycaemia-induced elevation of fibroblast growth factor basic, matrix metalloproteinase 9 and nephroblastoma overexpressed gene proteins, implicating a protective role of PACAP in vascular complications of diabetes (94). PACAP deficient mice are susceptible to develop a systemic form of senile apolipoprotein IV-predominant amyloidosis, characterized by typical perivascular deposits in most organs (75, 138), pointing to the role of PACAP in age-related vascular changes. This has been also confirmed in a study where angiogenic capacity was examined in cerebromicrovascular endothelial cells (197). In aged cells, expression of PACAP was decreased, associated with reduced capacity to form capillary-like structures, impaired adhesiveness to collagen, and increased apoptosis (caspase3 activity). Overexpression of PACAP in aged endothelial cells resulted in increased tube-formation capacity. Treatment with recombinant PACAP also increased tube formation and inhibited apoptosis in aged cells. In young cerebromicrovascular endothelial cells, knockdown of endogenous PACAP expression impaired tube formation capacity, mimicking the aging phenotype (197).

PACAP and its receptors occur in the heart and the neuropeptide exerts various different cardiac functions. Several studies examined the potential effects of PACAP in the cardiovascular system. The presence of this polypeptide and its PAC1 receptor have been demonstrated in cardiomyocyte cell cultures, mouse heart tissue and also in human heart samples (150, 198–200). PACAP has direct positive chronotrop, inotrop, dromotrop, and vasodilatator effects and exerts robust cardioprotective actions due to its antiapoptotic and antioxidant properties (201). The cardioprotective effects of PACAP were first identified in cardiomyocyte cell cultures against different ischemia/reperfusion injuries. In these experiments, exogenous PACAP treatment lead to decreased level of pro-apoptotic factors (Bad, caspase-3) and elevated levels of anti-apoptotic proteins (cleaved caspase-8, Bcl-xl) inducing significantly increased cell viability (50, 51). Moreover, several *in vitro* experiments have already proven that PACAP provides effective protection against oxidative stress induced apoptosis due to higher Bcl-2 and phospho-Bad expression and lower caspase or Jun N-terminal kinase and p38 mitogen activated protein (MAP) kinase activation (51–53).

Besides *in vitro* researches, different animal studies also examined the cardioprotective effects of PACAP. Alston et al. detected significantly higher endogenous PACAP levels and mRNA expression in mice after myocardial infarction (202). Furthermore, examining doxorubicine induced cardiomyopathy, significantly more severe DNA damage and apoptotic cell death were detected in PACAP-deficient mice compared to wild types (95). In another study, PACAP treatment had protective effect against mitoxandron induced cardiotoxicity (96). PACAP attenuated body weight reduction of mice, prevented mitoxantrone-induced left ventricular dilation resulting in diminished functional deterioration shown by the ejection fraction and fractional shortening (96). A recent study provided both *in vitro* and *in vivo* evidence that PACAP attenuates radiation-induced heart disease, a common consequence of thoracic irradiation therapy (97). PACAP enhanced viability and colony-forming efficiency, while reduced generation of reactive oxygen species in cardiomyocytes exposed to radiation. PACAP exposure suppressed myocardial apoptosis and G2/M arrest through blunting the radiation-induced down-regulation of Bcl-2, CyclinB1 and CDC2, and inhibiting the up-regulation of Bax (97). Pre-treatment with PACAP also protected mice from radiation-induced histological damage including myocardial apoptosis and fibrosis (97).

In addition to the several *in vitro* and animal experiments, a few human studies have also been performed that raise the possibility of PACAP being cardioprotective also in the human heart. Szanto et al. examined human right auricle tissue samples from coronary artery bypass or heart valve implantation surgery. They found significantly higher PACAP concentration in patients with ischemic heart disease compared to valvular abnormalities emphasizing the protective role of PACAP in ischemic heart diseases (150). Remarkable differences were detected in plasma PACAP levels of ischemic and primary dilated cardiomyopathy patients. A significant negative correlation was observed between the severity of ischemic heart failure and plasma PACAP levels suggesting that PACAP might play an important role in the pathomechanism and progression of ischemic heart failure (203). Lack of PAC1-mediated signaling has been shown to be associated with pulmonary hypertension and right heart failure in PAC1 deficient mice, indicating the crucial importance of PACAP in the maintenance of normal pulmonary vascular tone (151).

## Reproductive Organs

PACAP and its receptors have widespread and stage-specific occurrence in the gonads and their presence have been shown in several other reproductive organs (100, 204, 205). PACAP has been described as a follicle-surviving factor due to suppression of apoptosis in a dose-dependent manner in rat ovaries (206). Although PACAP has been detected in the human ovarian follicular fluid, its exact role in the human ovary is not known (207). In the testis, PACAP plays a role in spermatogenesis and is supposed to be involved in tumor growth (100, 208, 209). Interestingly, in spite of PACAP deficient mice displaying disturbed spermatogenesis and altered testicular immunity (100, 210), they also show delayed testicular aging supposedly due to the stimulatory effect of PACAP on testosterone production, which, in turn, accelerates aging due to increased oxidative stress (211). The involvement of PACAP has been described in numerous reproductive processes from fertilization to implantation (212–214). However, its direct cytoprotective effects have only been shown in a few cases. PACAP protects human trophoblast cells against oxidative stress, but has no effect against methotrexate-induced cell death and in contrast, it potentiates cell death in choriocarcinoma cells (205, 215, 216). Similar tumor cell growth inhibiting effects have been described in cervical cancer cells (217). These data demonstrate that while in many tissues PACAP is clearly a pro-survival factor, no such clear effect has been found in the reproductive tissues, where PACAP seems to play a more complex regulatory role in reproductive processes and during tumor growth.

## Other Cell Types

One study described that PACAP was able to attenuate apoptosis of human hypophysis adenoma cell line HP75 induced by transforming growth factor-β1 (218). In PC-3 human prostate cancer cells PACAP inhibited apoptosis induced by serum starvation (219). PACAP alone did not influence cell survival in cultured pinealocytes, but could attenuate the toxic effect of H_2_O_2_. However, co-incubation of pinealocytes with PACAP promoted survival only in the dark phase, PACAP during the light phase did not result in significant differences in the percentage of living cells. This suggests that the time of day can also influence the protective effects of PACAP (220). PACAP inhibited serum depletion-induced apoptosis of megakaryocytes via VPAC1 stimulation (221).

## Contradictory Aspects

Based on the studies summarized here PACAP is now considered as a general cytoprotective peptide. However, its cell survival-promoting effect depends on a number of factors. First of all, it depends on the type of cell and developmental stage of a cell type. Also, numerous other factors can influence whether PACAP acts as a pro-survival factor, has no effect, or even on the contrary, it induces cell death (222). For example, PACAP has been described to counteract the cytotoxic effects of cisplatin chemotherapy treatment in neurons, without affecting the toxic effects in ovarian cells, thereby not influencing the therapeutic effect of cisplatin on tumor cells (223). Glioblastoma cell invasion is inhibited by PACAP under low oxygen tension (224), while both pro- and anti-proliferative effects have been found in different glioblastoma cell lines (225, 226). As we summarized the cytoprotective effects of the peptide present in several cell types, PACAP has also been described to exert cytotoxic effects in high doses in certain tumor cells, like in retinoblastoma cells (227). In certain cells, neither pro- nor anti-survival effects could be found, like JAR cytotrophoblast cells with or without methotrexate treatment (205) or in HEP-G2 hepatocellular carcinoma cells subjected to oxidative stress (22). In contrast, in several other tumor cell line, PACAP has been shown to inhibit growth, like MCF-7 breast cancer cells (126), a negative regulator of cervical carcinoma as overexpression of the PACAP gene in cervical cancer cell lines lacking PACAP expression significantly inhibited cell growth and induced apoptosis (217), is a pro-apoptotic factor in choriocarcinoma cells (215), and inhibits the growth of myeloma, leukemia and medulloblastoma cells (228–230).

## Summary

In summary, PACAP has protective effects against various harmful stimuli in a wide range of tissues in the periphery. Numerous factors influence this protective effect, the detailed mapping of which awaits further investigatons.

## Author Contributions

DT and DR conceptualized the paper. DT and VV constructed the figure. AT, ES, TJ, GH, EF, BO, DS, GM, AD'A, and VD'A wrote parts of the review.

## Conflict of Interest

The authors declare that the research was conducted in the absence of any commercial or financial relationships that could be construed as a potential conflict of interest.
